# Oxidative Stress in Cell Death and Cardiovascular Diseases

**DOI:** 10.1155/2019/9030563

**Published:** 2019-11-04

**Authors:** Tao Xu, Wei Ding, Xiaoyu Ji, Xiang Ao, Ying Liu, Wanpeng Yu, Jianxun Wang

**Affiliations:** ^1^School of Basic Medical Sciences, Qingdao University, Qingdao, China; ^2^Center for Regenerative Medicine, Institute for Translational Medicine, College of Medicine, Qingdao University, Qingdao, China; ^3^Department of Comprehensive Internal Medicine, Affiliated Hospital, Qingdao University, Qingdao, China

## Abstract

ROS functions as a second messenger and modulates multiple signaling pathways under the physiological conditions. However, excessive intracellular ROS causes damage to the molecular components of the cell, which promotes the pathogenesis of various human diseases. Cardiovascular diseases are serious threats to human health with extremely high rates of morbidity and mortality. Dysregulation of cell death promotes the pathogenesis of cardiovascular diseases and is the clinical target during the disease treatment. Numerous studies show that ROS production is closely linked to the cell death process and promotes the occurrence and development of the cardiovascular diseases. In this review, we summarize the regulation of intracellular ROS, the roles of ROS played in the development of cardiovascular diseases, and the programmed cell death induced by intracellular ROS. We also focus on anti-ROS system and the potential application of anti-ROS strategy in the treatment of cardiovascular diseases.

## 1. Introduction

ROS refers to a group of small reactive molecules and is produced under both the normal life process and the various pathological conditions. ROS can function as a signaling molecule or a risk factor for the occurrence of diseases [1]. The levels of intracellular ROS are precisely regulated to limit it to a certain level. However, intracellular ROS can be damaged to the cell if ROS level is out of the normal range under the pathological conditions. The intracellular ROS is closely correlated with the pathogenesis of cardiovascular diseases, including the atherosclerosis, myocardial ischemia/reperfusion injury, myocardial hypertrophy, and heart failure [2]. However, the current therapeutic strategies to target the intracellular ROS are unsuccessful in the clinical trial of cardiovascular disease treatment. The reason of the failure is ascribed to the inability to clarify the specific roles of ROS and target the accurate ROS resources under different pathological conditions, and the nonspecific antioxidant approach cannot scavenge ROS properly and effectively [3]. Understanding the precise mechanism of ROS production, ROS-related signaling pathways, and the different roles ROS played under different pathological conditions is essential for increasing the chance of success during cardiovascular disease treatment. Moreover, cell death induced by ROS is closely related with the pathogenesis of cardiovascular diseases. Exploration of the mechanisms of cell death and the development of anticell death strategy will also provide opportunity for the cardiovascular disease treatment.

## 2. ROS Resources in the Cardiovascular Diseases

### 2.1. Excessive ROS in Vascular Dysfunction

Even though small amounts of intracellular ROS are continuously produced in cells, excessive generation of ROS, caused by pathological stimuli or the failure of ROS clearance system, is the major cause of various vascular dysfunctions. Accumulating evidences suggest that excessive ROS contributes to the altered vascular functions including endothelial dysfunction, vascular smooth muscle cell (VSMC) overgrowth, and structural remodeling. Moreover, oxidative stress could induce vascular inflammation and injury through activation of the transcription factors, upregulation of adhesion molecules, stimulation of chemokine production, and recruitment of inflammatory cells [4, 5]. Considering these important roles of ROS in the pathogenesis of vascular dysfucntion, a clear classification of the ROS resources and the roles it plays under different pathological conditions is urgently needed.

#### 2.1.1. ROS from the NADPH Oxidase Activity

**Figure 1:**
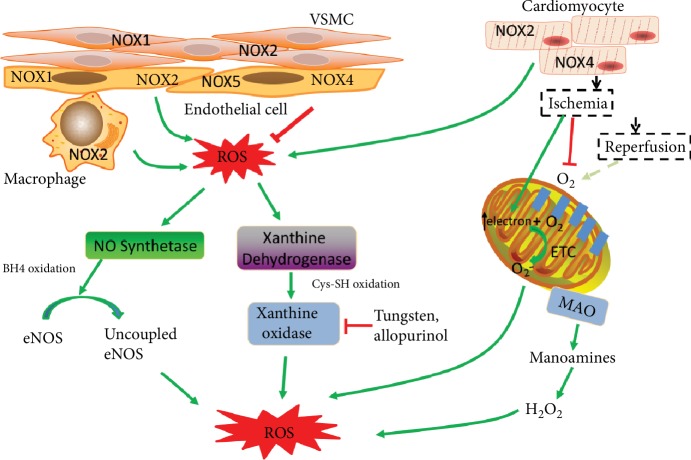
ROS resources during cardiovascular diseases. The NOX-derived ROS are the primary ROS resources. NOX1, NOX2, NOX4, and NOX5 are expressed in the endothelial cell. NOX1 and NOX2 are expressed in the VSMC. NOX2 and NOX4 are abundant in cardiomyocyte. The activity of NOX2 in the immune cells also contributes to the ROS production under pathological condition. NOX-derived ROS can uncouple the NO synthase and promote O_2_^−^ generation. The xanthine dehydrogenase is transformed into xanthine oxidase by oxidation which uses oxygen as an electron acceptor and produces ROS. Ischemia disrupts the oxygen supply and promotes the electron accumulation of electron transport chain. Reperfusion recovers the oxygen and promotes O_2_^−^ production. Monoamine oxidase (MAO) anchored on the mitochondrial outer membrane degrades the monoamines and produces H_2_O_2_.

Although the intracellular ROS comes from multiple sources, the activities of NADPH oxidases (NOXs) are the only primary ROS resources (Figure 1) [6, 7]. NOXs could generate a large burst of O_2_^−^ with NADPH serving as electron donor in the VSMC, endothelial cells, and fibroblasts [8]. Moreover, NOX-derived ROS can uncouple the NO synthase and promote O_2_^−^ generation through oxidative degradation of NO synthase cofactor, H4B. There are 5 different NOX isoforms identified until now [8, 9]. NOXs are expressed in a cell- and tissue-specific fashion and are differently regulated under various pathological conditions (Figure 1).

The NOX1, NOX2, NOX4, and NOX5 are all expressed in endothelial cells [10]. Other cell types in the vascular wall, including VSMCs and the immune cells, also express NOXs, which also contribute to ROS production under certain conditions (Figure 1) [11–13]. NOX2 is likely to be the most important ROS resource under pathological conditions while NOX4 plays a protective role in contrast through promoting NO bioavailability and suppressing cell death [14]. The remnant lipoprotein particles (RLPs) or oxLDL, which are the coronary risk factors and predictors of cardiovascular events, will increase NOX2 expression and the subsequent ROS production but have no effect on the expression of NOX4 in endothelial cells [15]. It is shown that NOX2 knockout mice protect endothelial cells from ROS damage in the aorta in the atherosclerosis model, suggesting that NOX2-deprvied ROS is the major cause of atherosclerosis [16]. Angiotensin II is a potent inducer of vascular ROS production and promotes the vessel dysfunction. Evidences showed that Ang II could increase the expression of NOX2 and promote the acute assembly of this oxidase complex in the endothelial cells. In contrast, NOX4 could antagonize Ang II-induced endothelial dysfunction. NOX4 knockout mice accelerate the aortic medial hypertrophy and cytokine production in the mouse model [17].

#### 2.1.2. Xanthine Oxidase

The activity of xanthine oxidase (XO) is another major source of intracellular ROS. There are two forms of XOs with different substrates. The dehydrogenase form uses both NAD+ and oxygen as an electron acceptor, with a preference to NAD+. The oxidase form of XO using the molecular oxygen as electron acceptor produces ROS without reducing NAD+. Evidences show that Ang II could increase the protein levels of XO. XO knockout dramatically decreases the ROS production during Ang II-induced vascular dysfunction, suggesting that XO activity is a major ROS resource under this condition [18]. NOX inhibition can prevent the Ang II-induced superoxide from XO, suggesting that the activation of XO by Ang II needs the activity of NOX [19]. Cytokines can also stimulate the expression of XO, and XO is involved in ROS production induced by vascular inflammation [20, 21]. The activity of endothelial XO is also observed to be increased in the coronary disease patients. All these evidences support the application of targeting XOs during the cardiovascular disease treatment.

The protective roles of XO inhibitors have been tested in the animal models of cardiovascular diseases. Tungsten, an inhibitor of XO, can prevent the development of atherosclerosis in ApoE^−/−^ mice [22]. Allopurinol, another inhibitor of XO, can attenuate endothelial dysfunction in HF patients [23]. In addition, the product of XO is a biomarker during the diagnosis of cardiovascular diseases [24]. These evidences suggest the important roles of XO in the vascular dysfunction, and targeting XO may represent an important way for disease treatment.

### 2.2. ROS in Cardiac Remodeling and Heart Failure

The cardiac pathological conditions including cardiomyocyte hypertrophy, dysregulation of cell death, and remodeling of the extracellular matrix contribute to the final heart failure. Accumulating evidences demonstrate that intracellular ROS and its related signaling pathways are actively involved in these cardiac functional abnormalities.

#### 2.2.1. ROS from Enzymatic Activity

NOXs are also involved in the myocardial ROS production process (Figure 1). Both in the experimental models of the left ventricular hypertrophy (LVH) and in the end-stage failing human myocardium, the increased NOX activity is found to be closely correlated with these pathogenic processes [25, 26]. NOX2 and NOX4 are abundantly expressed in the cardiomyocytes. In the mouse model of LVH induced by angiotensin II or atrial natriuretic factor, ROS production is inhibited and cardiac function is improved by NOX2 knockout, indicating that NOX2-derived ROS plays a critical role in Ang II-induced hypertrophy [27, 28]. However, cardiac pressure overload-induced hypertrophy cannot be inhibited by NOX2 knockout, suggesting that NOX2 is not important in this process. In contrast, NOX4 promotes the LVH induced by pressure overload [29, 30]. Adverse remodeling of the left ventricle caused by myocardial infarction will develop into the final chronic heart failure (CHF) in patients. ROS generated from the NOXs activates the matrix metalloproteinase, which drives matrix turnover and promotes the left ventricle dilatation [31]. The NOX-derived ROS activity also plays an important role in myocardial infarction both in the mouse model and in the patients [25, 32]. The activity of xanthine oxidase (XO) also contributes to the cardiac adverse left ventricle remodeling after myocardial infarction in the mouse model. Inhibitor of XO improves the cardiac function in the mouse model of myocardial infarction (MI) [33]. Monoamine oxidase (MAO) is another ROS resource anchored in the outer membrane of mitochondria [34]. The activity of MAO increases the levels of H_2_O_2_ both in the mitochondria and the cytosol (Figure 1), which impairs the autophagy process, leading to the accumulation of damaged organelles and the final myocardial necrosis [35]. MAO is also involved in the pathogenesis of heart failure and myocardial ischemia/reperfusion injury [36, 37].

#### 2.2.2. ROS from Mitochondria

The activity of mitochondria electron transport chain (ETC) produces ATP for the cellular energy demand with the oxygen as the electron acceptor (Figure 1), which is another ROS resource [38]. Moreover, mitochondrial dysfunction during the pathological process can lead to the ROS burst which activates multiple cell death signaling pathways. Evidences show that mitochondrial ROS is produced in both the ischemia stage and the reperfusion stage during myocardial ischemia/reperfusion injury. Ischemia disrupts the oxygen supply and induces the collapse of electron transport chain (ETC), with the accumulation of electron. The O_2_ and ATP depletion during ischemia also sets the condition for the ROS production during reperfusion stage [39, 40]. Reestablishment of oxygen at the reperfusion stage acutely increases the generation of ROS, which leads to subsequent myocardial cell death [41]. Moreover, mitochondrial ROS will lead to the inactivation of iron-sulfur (Fe-S) centers, releasing free iron and leading to subsequent lipid oxidation through Fenton reaction [42].

## 3. ROS and Programed Cell Death

**Figure 2:**
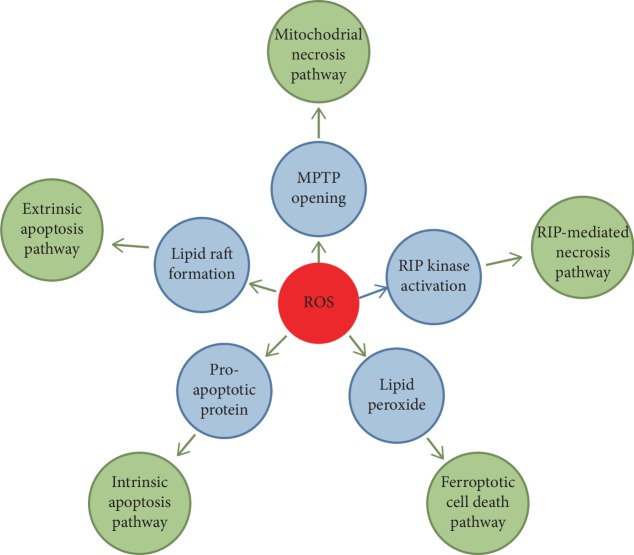
Schematic diagram of programmed cell death during ROS-induced myocardial injury.

A direct outcome of excessive ROS production is the induction of cell death, which is the major cause of various cardiovascular diseases under different pathological conditions [43]. Programmed cell death is the important therapeutic target during disease treatment as it is regulated by the gene products [44]. Apoptosis is the first established programmed cell death and has been studied intensively in the past 2 decades. In addition to the apoptotic cell death, other modes of programed cell death have recently been identified and are demonstrated to contribute to the pathogenesis of cardiovascular diseases (Figure 2) [45]. Understanding the roles of different cell death processes in cardiovascular diseases and the underlying signaling pathways will improve the therapeutic strategy.

### 3.1. Apoptosis

Apoptosis is the firstly coined and mostly studied programmed cell death process. Numerous studies show that apoptosis contributes to both the acute loss of cardiomyocytes in myocardial ischemia/reperfusion injury and the chronic loss of cardiomyocytes in the chronic heart failure [46]. ROS is closely related with cardiomyocyte apoptosis. Both the extrinsic death receptor pathways and the intrinsic mitochondrial pathways are involved in the myocardial apoptosis induced by ROS. The binding of ligands with death receptor induces lipid raft formation and NOX assembly and activation, which leads to the ROS generation. ROS will promote the formation of lipid raft-derived signaling platforms, activating the death receptor-mediated apoptosis [47].

During the activation of the intrinsic mitochondrial apoptosis pathways, excessive ROS promotes the permeabilization of the mitochondrial outer membrane through activation of the proapoptotic Bcl-2 superfamily proteins [48]. Increased mitochondrial permeability will lead to the release of apoptosis activators, triggering the activation of apoptosis [49]. What is more, ROS can induce apoptosis through the activation of apoptosis initiation signaling pathways or inhibition of the protective mechanisms of the cell [50, 51].

Although the myocardial apoptosis signaling pathways have been intensively studied and therapeutic targeting of apoptotic pathways shows potential in the treatment of heart failure, it is still not convincing to say that the inhibition of apoptosis can efficiently prevent heart failure in the patients. One reason may be that inhibition of apoptosis may activate the necrotic cell death, which is a major cell death process during the heart failure. Another important thing may be that it is important to initiate treatment at the most suitable time. For example, cell death may occur in a certain stage, such as the beginning of reperfusion stage.

### 3.2. Myocardial Necrosis

It is considered that myocardial necrosis has a stronger effect on the loss of cardiomyocyte than apoptosis [52]. Recent evidences demonstrate that some necrosis can also be regulated by multiple signaling pathways rather than a passive cell death process [53]. There are two major signaling pathways regulating the myocardial necrosis, the RIP-mediated necrosis pathway and the mitochondrial necrosis pathway [54]. The burst of intracellular ROS during the myocardial I/R injury through XO activity or mitochondria ROS formation leads to myocardial necrosis and promotes myocardial damage [55]. The role of ROS in the induction of myocardial necrosis can also be demonstrated from several recent works. Wang et al. showed that ROS will elevate the protein levels of RIP1 and RIP3, promoting the H_2_O_2_-induced necrosis in H9c2 cells. These results are also confirmed in the mouse model of ischemia/reperfusion injury [56]. Another study by Zhang et al. demonstrates that RIP3 can promote the activity of CAMK II under the doxorubicin or H/R-induced oxidative stress through phosphorylation and oxidation of CAMK II. CAMK II activation increases the mitochondrial calcium, which induces myocardial necrosis [57]. Evidences show that MPTP (mitochondrial permeability transition pore) opening at the beginning of the reperfusion stage contributes to almost 50% of the infarct size while ROS is the potent inducer of MPTP. Cypd is the most important component of MPTP. Cypd-deficient mice subject to ischemia/reperfusion injury lead to the smaller infarcted size than the wild-type mice. The inhibitor of Cypd, cyclosporin A (CsA), can also decrease infarcted size in the in vivo mouse model [58]. Another study shows that apoptosis repressor with card domain (ARC) can inhibit MPTP opening by interacting with Cypd and blocking the MPTP complex assembly. Under the oxidative condition, p53 is upregulated and represses the expression of ARC at transcriptional level, which releases the Cypd from binding with ARC and promotes the opening of MPTP [59].

Nec-1 is a specific RIP1 inhibitor and could markedly reduce infarct size in the cardiac ischemia/reperfusion injury. Nec-1 can also prevent the cardiac adverse remodeling after ischemia/reperfusion in the mouse model [60]. However, Nec-1 has been reported to cause cell death in some cases during the clinical trial. Fortunately, the Nec-1 analogue, Nec-1s, had been developed and the promotion of cell death is not observed [61]. There are also some other inhibitors of RIP-1 that have recently been developed. GSK963 is a new RIP1 inhibitor and is more effective than Nec-1 in inhibiting RIP1-dependent cell death. The clinical application of CsA is validated only in a small number of patients, and these results are doubted by several studies and a larger clinical trial is required for the further confirmation [62, 63]. A present work reports that a modified mtCsA (mitochondria-targeted CsA) with a much-improved Cypd binding affinity yields better cardioprotective role than CsA in a mouse model of I/R [64]. Although these necrosis inhibitors show great potential in the treatment of heart diseases, its clinical significance is still under debate and needs to be further verified [65].

### 3.3. Autophagic Cell Death

A basal level of autophagy is essential for removal and renewal of dysfunctional organelles and damaged proteins. The cardiomyocytes also depend on autophagy to maintain intracellular homeostasis. Autophagy has also been linked to cardiovascular diseases under the oxidative stress [66]. Intense investigation of the role of autophagy under pathological conditions has currently been carried out. However, evidences show that autophagy seems to play dual roles in cardiovascular diseases caused by excessive ROS. On one side, there are numerous evidences showing that autophagy could protect the cardiomyocytes from injury in the cardiac dysfunction. During the ischemia/reperfusion injury, researchers find that autophagy flux is markedly reduced in cardiomyocytes with downregulation of LAMP2 and BECN1 [67, 68]. Upregulation of autophagy through rapamycin treatment can attenuate myocardial ischemia/reperfusion injury [69, 70]. In the models of doxorubicin cardiotoxicity, doxorubicin blocks cardiomyocyte autophagic flux accompanied by robust accumulation of undegraded autolysosomes. This impaired autophagy process is harmful to the cardiomyocytes, and clearance of these undegraded autolysosomes will relieve the doxorubicin cardiotoxicity [71]. On another side, there are also evidences supporting the negative roles of autophagy in the survival of cardiomyocytes under oxidative stress from several studies. In the high-glucose-induced ROS generation and cardiomyocyte dysfunction, autophagy flux is also inhibited. However, researchers show that this autophagy inhibition is only an adaptive response. Conversely, rapamycin treatment or BECN1 or ATG7 overexpression could increase autophagy and promote cardiomyocyte death induced by high glucose [72]. In another study, Liu et al. found that autophagy promotes the H_2_O_2_ and myocardial ischemia/reperfusion-induced cardiomyocyte injury which is attenuated by inhibition of autophagy through a LncRNA, a cardiac autophagy inhibitory factor (CAIF) [73]. These evidences support the roles of autophagy in promoting myocardial cell death. In summary, the role of autophagy in cardiomyocyte death depends on the specific situation. Moderate autophagy may promote survival by removing damaged organelles caused by ROS and recycling macromolecules to maintain energy levels. However, prolonged ischemia and subsequent reperfusion result in excessive autophagy which contributes to the self-digestion and ROS production.

### 3.4. Ferroptosis

Ferroptosis is a newly corned programmed cell death process. The distinct feature of ferroptosis is the iron-dependent lipid peroxide accumulation [74]. Ferroptosis also participates in the pathogenesis of cardiovascular diseases. In an ex vivo heart model of ischemia/reperfusion injury, inhibition of ferroptosis by treatment with glutaminolysis inhibitor compound 968 or iron chelator DFO could improve the heart function and reduce the infarct sizes [75]. Doxorubicin is a traditional antitumor drug whose clinical application is limited by its cardiotoxicity. Doxorubicin's cardiotoxicity is usually attributed to its role in myocardial apoptosis initiation [76]. However, the recent work by Fang et al. shows that ferroptosis occurs in the doxorubicin-induced cardiomyopathy. In this study, researchers find that doxorubicin can elevate the mitochondria iron level and cause mitochondrial lipid peroxide accumulation, which promotes an oxidative stress and the subsequent ferroptotic cell death [77]. Although the roles of ferroptosis have been partially delineated in the cardiomyocytes, the regulatory mechanism of ferroptosis remains largely unknown. Myocardial ferroptosis may occur in some other human pathological conditions. For example, high levels of heme iron in the diet will increase the iron levels in the circulation, which can be taken up by the cardiomyocyte. A fraction of the circulating iron is redox-active, and the excessive uptake of iron may contribute to oxidant-mediated ferroptotic cell death [78]. Until now, the cardioprotective role of ferroptosis is only tested in a mouse model. The application of ferroptosis inhibitors in the treatment of cardiovascular diseases needs to be further explored.

## 4. Anti-ROS Systems and Clinical Application

### 4.1. Endogenous Antioxidant System

#### 4.1.1. GSH-Linked Enzymatic Defense Systems

**Figure 3:**
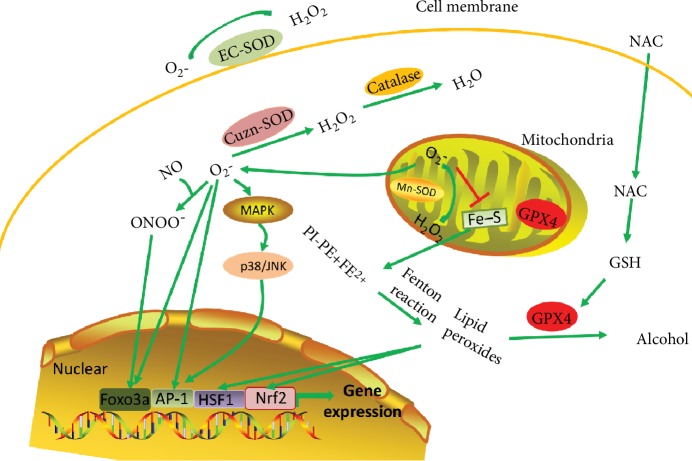
The anti-ROS system. SOD could transform the O_2_^−^ into H_2_O_2_ and then could be reduced to water by catalase. There are three forms of SODs: EC-SOD (extracellular matrix), CuZn-SOD (cytoplasm), and Mn-SOD (mitochondria). Mitochondrial O_2_^−^ inactivates iron-sulfur (Fe-S) centers and releases free iron, leading to subsequent lipid oxidation through Fenton reaction. GPX4 could catalase the lipid peroxides into alcohol. Antioxidant NAC could promote the synthesis of GSH which is a cofactor of GPX4. ROS will also activate a series of transcription factors (AP-1, Foxo3a, HSF-1, and Nrf2) whose target genes defend against the oxidative stress-related damage.

To protect against the damaging effects of ROS, cells have developed multiple antioxidant systems to guarantee the timely removal of ROS. One of the most important is the tripeptide glutathione, GSH, and the GSH-linked enzymatic defense systems. GSH functions as a cofactor for the GSH-peroxidase families. GPX4 is one of the GSH-peroxidases and can interact with lipid hydroperoxides efficiently and catalyze the degradation of peroxides. The mitochondrial GPX4 is the first defense system to avoid ROS damage. The deletion of mitochondria GSH is closely correlated with the ROS toxicity-related cell death process (Figure 3). Although GSH also exists in the cytosol, it seems that cytosolic GSH is less important than that in the mitochondria [79]. Overexpression of mitochondrial GPX4 attenuates myocardial ischemia/reperfusion injury [79]. Recent experiments have also shown that GPX4 is an inhibitor of ferroptosis through the clearance of lipid peroxides (Figure 3). As ferroptosis also promotes the pathogenesis of cardiovascular diseases, the inhibition of ferroptosis by GPX4 will improve the cardiac function under certain pathological conditions [80]. GPX4 can also maintain the vascular homeostasis through its ROS clearance activity. Evidences show that overexpression of GPX4 could suppress the atherosclerosis in ApoE^−/−^ mice [81].

#### 4.1.2. Superoxide Dismutase (SOD) and Catalase

SODs are another antioxidant systems and protect against superoxide-mediated cytotoxicity. There are three forms of SODs that have different intracellular localization. O_2_^−^ is the main form of superoxide and is generated by complex I and complex III. SOD could transform the O_2_^−^ into H_2_O_2_, which is a relatively stable and diffusible compared with many other ROS, and then could be reduced to water by catalase (Figure 3). Another role of SODs is to maintain the NO level in the endothelial cell which plays a major role in maintaining the basal vasodilator tone of the vessel. Superoxide could react with NO efficiently and make NO unavailable. Moreover, reaction of NO with superoxide will produce peroxynitrite, a potent oxidant with potential cytotoxicity (Figure 3) [82]. Thus, timely clearance of superoxide by SODs determines the bioactivity of NO. Overexpression of SODs in the mouse model of I/R exposed to ischemia/reperfusion injury can decrease levels of superoxide and infarcted size and improve cardiac function. Similarly, specific overexpression of catalase in cardiomyocyte is also found to reduce the adverse remodeling and prevent heart failure in a mouse model of dilated cardiomyopathy. Overexpression of SODs or catalase can also retard the atherosclerosis in the ApoE^−/−^ mice [83]. All these results demonstrated the protective role of SODs against the ROS-induced cardiovascular diseases.

#### 4.1.3. The Thioredoxin (Trx) System

The thioredoxin (Trx) system defends against oxidative stress through its disulfide reductase activity. The removal of ROS though the Trx system also plays important roles in defensing the oxidative stress under pathological conditions. Overexpression of thioredoxin 2 attenuates Ang II-induced vascular dysfunction [84]. Loss of mitochondrial Trx reductase causes the inflammation and endothelial dysfunction [85]. Thioredoxin is also actively involved in the protection of cardiac function. Evidences show that the thioredoxin activity, which is regulated by thioredoxin-interacting protein, controls cardiac hypertrophy [86]. Inactivation of nitrative thioredoxin promotes cardiomyocyte injury induced by high glucose and cardiac ischemia/reperfusion [87]. Thioredoxin activity can also be inhibited by methylglyoxal, aggravating cardiomyocyte ischemia/reperfusion injury [88]. All these evidences show the close correlation of the thioredoxin with the cardiovascular homeostasis.

#### 4.1.4. Transcription Factors Activated in Defensing the Oxidative Stress

Cells could also activate a series of transcription factors whose target genes strongly defends against the oxidative stress through different ways. These transcriptional factors include the AP-1, HSF1, Nrf2, and the FOXO3a (Figure 3). These transcriptional factors are very sensitive to the intracellular oxidative stress and can drive the expression of target genes rapidly which will protect the cells from damage or kill the cells to avoid further damage. Ap-1 is a leucine zipper transcription factor and is activated by the H_2_O_2_ or JNK [89, 90]. AP-1 can bind to the promoter region of SOD in response to the oxidative stress and promote the expression of SOD to defend against the oxidative stress damage [91, 92]. However, the role of AP-1 during the pathogenesis of cardiovascular diseases is still under debate. Heat shock transcription factor 1 (HSF1) could translocate into the nuclear and assemble into a homotrimer, which can bind to the DNA and drive the expression of heat shock proteins (HSPs), in response to the oxidative stress [93]. The HSPs are considered to protect the cardiomyocytes from oxidative damage in the ischemic cardiac diseases [94, 95]. FOXO3a is another transcriptional factor exerting a strong protective role in the ROS-related cardiovascular diseases. FOXO3a can increase the expression of p27 and inhibit smooth muscle cell proliferation which promotes the vascular dysfunction under oxidative stress [96]. FOXO3a is the most abundant isoform expressed in the heart among the Foxo transcription factors. FOXO3a knockout will exacerbate the myocardial ischemia/reperfusion injury with decreased expression of catalase or SOD [97]. FOXO3a also participates in myocardial autophagy regulation. Evidences showed that overexpression of FOXO3a will increase the autophagy level and prevent the cardiac hypertrophy [98]. Nrf2 has recently been found to prevent the ferroptotic cell death. One of the important targets of Nrf2 is the GSH-linked enzyme, GPX4, which is an active ROS antagonist as mentioned above [99]. Nrf2 can also regulate the iron metabolism genes and attenuate ferroptosis through preventing free iron availability.

### 4.2. Exogenous Antioxidant Strategy

N-acetylcysteine (NAC) is a potent antioxidant which promotes the synthesis of GSH, attenuates oxidative stress, and prevents cell death. Evidences from the animal models show that NAC can attenuate cardiac injury, prevent cardiac fibrosis and remodeling, and improve the cardiac function during the heart failure [100]. Moreover, NAC can also inhibit maladaptive autophagy, which promotes the pathogenesis, in pressure overload-induced cardiac remodeling in rats [101]. The role of NAC has also been validated in the myocardial infarction patients, although these results need to be further explored in a larger population [102]. Vitamin E is another antioxidant with the clinical potential to the treatment of cardiovascular diseases. Evidences from several experimental animal models show that vitamin E treatment is demonstrated to improve cardiac function [103]. However, vitamin E fails to prevent the atherosclerotic disease in the clinical trial. What is more, vitamin E is also proved to have no effect on both the acute myocardial injury and chronic heart failure patients [104, 105]. Recently, researchers found that high levels of NAD+ could improve cardiac function in a mouse model of heart failure. The role of NAD+ in defensing oxidative stress is also validated in several other works, showing the potential application in the anti-ROS strategy [106].

However, the inappropriate application of antioxidant strategy is also harmful to our body. ROS may function as a second messenger and participate in cell signaling transduction and redox regulation. These signalings mediated by mild ROS may even benefit cellular repair processes and improve protective systems [107]. However, the antioxidants cannot distinguish among the ROS with a beneficial physiological role and those that cause oxidative damage to the cell. The antioxidant may clear the most harmful ROS while on another side leave not enough ROS for their useful purposes. The direct outcome of anomaly low ROS may interfere with the immune system and essential defensive mechanisms for removal of damaged components of the cell, including those that are precancerous [108]. Thus, the overtaking of antioxidant may be harmful to our body.

### 4.3. Anti-ROS Strategy in Clinical Trial

In the clinical trials, researchers get disappointing results in cardiovascular disease treatment through oxidative stress inhibition strategy. The reason for these failure remains largely unknown. One reason may be that the inadequate understanding of the ROS production stage and the myocardial injury during different pathological conditions. In the myocardial ischemia/reperfusion injury, the ROS is largely produced at the beginning of reperfusion stage and mass myocardial cell death accrued at this stage. However, the current antioxidant therapies are not able to combat these ROS and cell death. Another reason may be that the ROS resources during the pathogenesis of heart failure are more complex than we now know. There are more complex ROS producers functioning in different pathological conditions. For example, oxypurinol, inhibitor of xanthine oxidase, has been shown to improve heart failure in a specific subset of patients with elevated uric acid. Thus, in these patients, targeting the xanthine oxidase may represent a better therapeutic strategy [109].

## 5. Conclusion

Excessive ROS production is the major cause of oxidative stress and cardiovascular diseases. Understanding the ROS production processes and the mechanism of anti-ROS systems in the cell will benefit the clinical practice in the treatment of cardiovascular diseases. Although the current antioxidants seem unsuccessful in the treatment of cardiovascular diseases, anti-ROS strategy still represents the most important ways for cardiovascular disease treatment. Efforts are still needed to illustrate the mechanisms of ROS production and how ROS promotes the pathogenesis of cardiovascular diseases under different conditions. Intracellular ROS is closely correlated with the myocardial cell death process, which takes an active role in the pathogenesis of cardiovascular diseases. Until now, there are several programmed cell death processes that are identified. However, whether all these cell death processes contribute to the pathogenesis of cardiovascular diseases and the major cell mode under different conditions in cardiovascular diseases are still needed to be illustrated. The revelation of the roles of different modes of cell death plays in the cardiovascular diseases will provide the precise drug target during disease treatment. Moreover, the exploration of ROS-related cell death signaling pathways will also help to develop the therapeutic strategy and protect cell death from oxidative stress.
